# Alleviation of Drought Stress by Hydrogen Sulfide Is Partially Related to the Abscisic Acid Signaling Pathway in Wheat

**DOI:** 10.1371/journal.pone.0163082

**Published:** 2016-09-20

**Authors:** Dongyun Ma, Huina Ding, Chenyang Wang, Haixia Qin, Qiaoxia Han, Junfeng Hou, Hongfang Lu, Yingxin Xie, Tiancai Guo

**Affiliations:** 1 National Engineering Research Center for Wheat, Agronomy, Henan Agricultural University, Zhengzhou 450002, China; 2 The National Key Laboratory of Wheat and Maize Crop Science, Henan Agricultural University, Zhengzhou 450002, China; 3 The Collaborative Innovation Center of Henan Food Crops, Henan Agricultural University, Zhengzhou 45002, China; Institute of Genetics and Developmental Biology Chinese Academy of Sciences, CHINA

## Abstract

Little information is available describing the effects of exogenous H_2_S on the ABA pathway in the acquisition of drought tolerance in wheat. In this study, we investigated the physiological parameters, the transcription levels of several genes involved in the abscisic acid (ABA) metabolism pathway, and the ABA and H_2_S contents in wheat leaves and roots under drought stress in response to exogenous NaHS treatment. The results showed that pretreatment with NaHS significantly increased plant height and the leaf relative water content of seedlings under drought stress. Compared with drought stress treatment alone, H_2_S application increased antioxidant enzyme activities and reduced MDA and H_2_O_2_ contents in both leaves and roots. NaHS pretreatment increased the expression levels of ABA biosynthesis and ABA reactivation genes in leaves; whereas the expression levels of ABA biosynthesis and ABA catabolism genes were up-regulated in roots. These results indicated that ABA participates in drought tolerance induced by exogenous H_2_S, and that the responses in leaves and roots are different. The transcription levels of genes encoding ABA receptors were up-regulated in response to NaHS pretreatment under drought conditions in both leaves and roots. Correspondingly, the H_2_S contents in leaves and roots were increased by NaHS pretreatment, while the ABA contents of leaves and roots decreased. This implied that there is complex crosstalk between these two signal molecules, and that the alleviation of drought stress by H_2_S, at least in part, involves the ABA signaling pathway.

## Introduction

Wheat (*Triticum aestivum* L.) is one of the most widely grown crops in the world, and it provides 20% of food calories to much of the world’s population. Plants are often subjected to periods of environmental stress during their life cycles, and drought is a one of the biggest factors threatening wheat yield in the world [1]. Drought stress can adversely affect crop growth and cause a reduction in plant leaf area [2], a decrease in photosynthesis [3], stem elongation, and stomatal movement [4]. According to Kettlewell, drought is a major cause of economic loss to the world's wheat growers, estimated at US$20 billion in 2000 [5]. In the coming decades of the 21th century, climate change will increase the chance of severe drought in many regions of the world. Thus, understanding the modes of action of exogenous substances that can improve drought tolerance in wheat could help alleviate the negative effects of drought and increase grain yield.

Hydrogen sulfide (H_2_S) is well known as an environmental toxin by virtue of its unpleasant odor of rotten eggs. Recent studies have suggested that hydrogen sulfide (H_2_S) is the third gaseous mediator after nitric oxide and carbon monoxide in mammals, and plays an important role in various biological processes such as smooth muscle relaxation, vasorelaxation, insulin signaling, and angiogenesis [6,7]. There is now considerable evidence to show that H_2_S may play a critical role in physiological and metabolic processes in plants [8, 9]. Several studies have demonstrated that exogenous application of an H_2_S donor (NaHS) can enhance resistance and/or tolerance to abiotic stress in higher plants. Li and Jin reported that NaHS alleviated the heat-induced decreased survival rate in cultured tobacco suspension cells [10]. NaHS pretreatment can also up-regulate the relative activities of antioxidant enzymes such as SOD and CAT in barley seedlings to alleviate oxidative damage caused by Al exposure [11]. H_2_S has also been found to participate in plant drought tolerance/resistance by increasing antioxidant enzyme activities [12], inducing stomatal closure [13], and up-regulating the transcript levels of genes involved in the ascorbic acid—glutathione cycle [14].

Abscisic acid (ABA) is a plant hormone that is involved in many aspects of plant growth and development throughout the plant life cycle, and ABA is regarded as a signal that can transmit drought information when plants suffer drought stress. The ABA biosynthetic pathway in higher plants has been fairly well characterized [15]. The oxidative cleavage of 9-cis-epoxycarotenoid to produce xanthoxin, catalyzed by 9-cis-epoxycarotenoid dioxygenase (NCED), is regarded as the rate-limiting step in ABA biosynthesis [16]. Most of the enzymes involved in the pathway leading to the biosynthesis of ABA have been determined; examples are zeaxanthin epoxidase (ZEP), abscisic aldehyde oxidase (AAO), and short-chain dehydrogenase (SDR). The ABA level in plants is controlled by a balance between the rates of ABA biosynthesis and catabolism. ABA 8′-hydroxylation (8′-OH) is a key step in the major route in ABA catabolism in several plant species [15]. ABA can be inactivated by conjugation with glucose [17], but inactive ABA can then be reactivated by β-glucosidases (GLU) [18]. The other proteins involved in the ABA pathway include an ABA receptor, the H subunit of Mg-chelatase (CHLH), and the regulatory component of ABA (RCAR).

H_2_S has been suggested to be the third member of the class of gaseous signalling molecules. Garcia-Mata and Lamattina [19] suggested that H_2_S might be involved in the ABA signal to induce stomatal closure in A. *thaliana* and *Vicia faba*. Li and Jin showed that H_2_S at least partially mediated the acquisition of heat tolerance induced by ABA in tobacco [10]. Although it has been well documented in previous reports that exogenous application of NaHS (an H_2_S donor) can improve the resistance or tolerance of plants to environmental stress, the underlying molecular mechanisms of the interplay between H_2_S and ABA are not well characterized. In this paper, wheat plants pretreated with NaHS were grown under normal and drought stress conditions. The expression profiles of ABA metabolic pathway genes in wheat leaves and roots were investigated, and the accumulation of ABA and H_2_S were also compared in order to gain an understanding of the role of ABA in H_2_S-mediated mitigation of drought stress.

## Material and Methods

### Plant material and chemical treatments

The common wheat (*Triticum aestivum* L.) cultivar ‘Yumai49-198’ was used in our experiments. Sodium hydrosulfide (NaHS, Sigma) was used as hydrogen sulfide (H_2_S) donor according to Hosoki et al. [7]. Wheat seeds were surface sterilized in 70% alcohol for 5 min, treated with 0.1% HgCl for 15 min, and washed six times (2 min each) in distilled water. The seeds were then germinated in Petri dishes (diameter 12 cm) placed in a temperature-controlled chamber (Ningbo Jiangnan Technology Co., China) at 25°C for 3 d. The germinated seeds were then shifted to a temperature-controlled chamber with a 16 hr/8 hr light/dark cycle (250 μmol m^−2^s^−1^), 25/15°C (light/dark), and 60/75% relative humidity. Seedlings were watered daily with appropriate volumes of Hoagland’s solution until the two-leaf stage. Based on preliminary experiments, 500 μM NaHS was selected as the treatment concentration in this study. At the two-leaf stage, the wheat seedlings were divided into three groups; (1) well-water control (CK), (2) PEG treatment (PEG), and (3) PEG combined with NaHS pretreatment (NaHS+PEG). The NaHS pretreatment was performed by treating seedling with NaHS solution for 48 h, and the solution was changed once at 12 h. After pretreatment, the seedlings in the PEG and NaHS+PEG groups were irrigated with PEG6000 (20%) solutions to artificially induce drought stress, and the stress lasted for 7 days. The PEG solution was changed once every two days. Wheat leaves and roots were collected for physiological assays every day after the treatments. Each treatment was replicated three times, and each replicate consisted of 100 seeds.

Additionally, a supplementary experiment was conducted to investigate the effect of ABA on the H_2_S content in leaves and roots. At the two-leaf stage, wheat seedlings were treated with exogenous ABA before being subjected to drought stress. The leaves and roots were collected after drought stress treatments and used for H_2_S content analysis.

### ABA, H_2_S, and leaf relative water contents

H_2_S concentration was measured according the method described by Chen et al. [20]. The H_2_S concentrations were determined by absorbance at 412 nm and are expressed as μmol/g fresh weight (FW). The quantitative measurement of ABA was carried out via an enzyme linked immuno-sorbent assay (ELISA) according to the methods of Guóth et al. [21]. ABA concentration in wheat samples is expressed as ng/g fresh weight. The samples for measuring leaf relative water content (RWC) were weighed immediatedly as fresh weight (FW), then sliced into 6-cm sections and soaked in distilled water for 24 hours at 4°C in the dark. The leaves were then removed from the water, and the surface water was blotted off the leaves and the turgid weights (TW) were recorded. Samples were then dried in an oven at 70°C to constant weight and the dry weight (DW) of each was recorded. The leaf relative water content was calculated using the following formula:

$$\text{RWC}\left(\text{\%}\right)=\left[\frac{\text{FW}-\text{DW}}{\text{TW}-\text{DW}}\right]\times 100$$

### Lipid Peroxidation and Hydrogen Peroxide

Malondialdehyde (MDA) content was measured by the procedures described by Wang et al. [22]. Tissue samples (0.5 g) were homogenized in 4.0 mL of 10% trichloroacetic acid (TCA) and centrifuged at 10000 × g for 10 min at 4°C. The supernatant fraction was mixed with 2 mL 20% TCA containing 0.5% thiobarbituric acid (TBA). The mixture was heated at 90°C for 20 min, cooled, and then centrifuged at 10000 × g for 5 min. The absorbance was recorded at 532 nm and the value for non-specific absorption at 600 nm was subtracted.

For determination of H_2_O_2_ concentrations, samples (0.1 g) were homogenized on ice in 0.1% (w/v) TCA. The homogenate was centrifuged at 10000 ×g for 15 min at 4°C, and a 0.5 mL sample of the supernatant was combined with 0.5 mL 10 mM potassium phosphate buffer (pH 7.0) and 1 mL of 1 M KI. The absorbance of the assay mixture was read at 390 nm and the content of H_2_O_2_ was calculated based on a standard curve of known H_2_O_2_ concentrations.

### Assays of SOD, CAT, and POD activities

The activities of superoxide dismutase (SOD), catalase (CAT), and peroxidase (POD) were assayed according to Garcia-Limones et al [23]. Samples were homogenized in ice-cold 50 mmol/L phosphate buffer (pH 7.8) by grinding in a mortar and pestle with liquid nitrogen. For each sample, the homogenate was centrifuged at 10000 × g at 4°C for 10 min, and the supernatant was then used for enzyme activity measurements. CAT activity was determined spectrophotometrically by monitoring the decrease in absorbance at 240 nm. SOD activity was assayed by measuring its ability to inhibit the photochemical reduction of nitro-blue tetrazolium. POD activity was based on the oxidation of guaiacol using hydrogen peroxide, and the increase in absorbance at 420 nm was read.

### RNA extraction, primer design, and real-time PCR

Total RNA was isolated from leaf and root samples using TriZol Reagent (Invitrogen) according to the manufacturer’s instructions. Three PCR amplifications were performed per sample to obtain the average expression level and the standard. Gene expression analysis was performed using SYBR Premix ExTaq (Promega Biotechnology [Beijing] Co., Ltd.), and the experiments were performed according to the manufacturer’s instructions. The DNA primer pairs used for amplification of ABA biosynthesis genes (*TaZEP*, *TaNECD*, *TaAAO*, and *TaSDR*), genes for enzymes involved in ABA metabolism (*Ta8’-OH1*and *Ta8’-OH2*), genes for ABA activation (*TaGLU1* and *TaGLU4*), and ABA receptor genes (*TaRCAR* and *TaCHLH*), as well as reference sequence numbers, are listed in Table 1. All primers were validated for amplication of the expected DNA fragments by cloning and sequencing the PCR products. The genes *TaZEP*, *TaNCED*, *Ta8’-OH1*, *Ta8’-OH2*, *TaGLU1*, and *TaGLU4*, encoding the corresponding enzymes in wheat, were identified in the NCBI database (http://www.ncbi.nlm.nih.gov). No genomic sequences for wheat *TaAAO*, *TaSDR*, *TaRCAR*, and *TaCHLH* were found directly. We used the cDNA sequences of the rice *AAO*, *RCAR*, *CHLH*, and *SDR* genes (Genbank accession numbers XM_ 015774953.1, JX970836.1, EU569725.1, and NM_001058201.1, respectively) as queries in BLAST searches against the wheat EST database in Genbank. Gene sequences HX165241.1, HX200293.1, CJ710881.1, and CJ794880.1 that had high levels of sequence homology to the rice *AAO* (83%), *RCAR*(88%), *CHLH* (90%), and *SDR* (82%) genes were selected as the corresponding wheat genes. For real-time PCR, two housekeeping genes, β-actin and GAPDH, were used as reference genes to analyze the relative expression levels of candidate genes in the samples. Because the expression patterns of these targeted genes with two reference genes were similar, only the relative expression levels of the target genes with GDPAH as the reference gene are given in this paper (S1 File).

**Table 1:** Names and sequences of oligonucleotide primers used for gene amplification in this study.

Gene	Primer	Sequence (5’-3’)	Reference sequence
*TaZEP*	Zep-F	TTGGAATGCCTTTGATGC	AF384103.2
	Zep-R	GCTGGTTGTTTGCCTTGT	
*TaNCED*	Nced-F	CCTGCTGCCTCTTCTGCT	KP099105.1
	Nced-R	ACCAAGTGCTCTTCCGTCTC	
*TaAAO*	Aao-F	TTGGCGTTGTGATTGCTG	HX165241.1
	Aao-R	GCTCAAGGTTCTCGGTGCT	
*TaSDR*	Sdr-F	AGTCCTCAAACGCCTTCA	CJ794880.1
	Sdr-R	TACCTGGCAAGCGACGA	
*Ta8’-OH1*	Oh1-F	AAGCCGTCACCGAAGAGC	AB714577.1.
	Oh1-R	ACCCGCATCGTCTCCTG	
*Ta8’-OH2*	Oh2-F	ACAGGTGGGAGGTTGTTGGA	AB849504.1
	Oh2-R	CTTCGTCGTCGTAGTCGTCATC	
*TaGLU1*	Glu1-F	CACAGAAGAGCAAGGGAA	AB100035.1
	Glu1-R	AAGTGGAGGCACCAAATA	
*TaGLU4*	Glu2-F	TCTACCACTATGACCTCCCG	JN128604.1
	Glu2-R	TCTTCACCCTGTCTCCAAAC	
*TaRCAR*	Rcar-F	ATCATAACAGTCCACCCACAG	HX200293.1
	Racr-R	CACGGCCTCAACGAAGTA	
*TaCHLH*	Chlh-F	CCCAACAGGGAAGAACAT	CJ710881.1
	Chlh-R	CAGGATACTTGCCACCATT	
*Actin*	Actin-F	TTTGAAGAGTCGGTGAAGGG	AB181991.1
	Actin-R	TTTCATACAGCAGGCAAGCA	
*GAPDH*	Gap-F	ACCACTAACTGCCTTGCTCCT	AF251217.1
	Gap-R	GTGCTGCTTGGAATGATGTTG	

### Data Analysis

The data were analyzed and evaluated using Statistical Program for Social Science (SPSS) software, and the results are shown as means ± standard deviation. The LSD test was used to distinguish differences between mean values, and *p*<0.05 was considered to be statistically significant.

## Results

### NaHS pretreatment alleviates drought stress during growth of wheat seedlings

**Fig 1:**
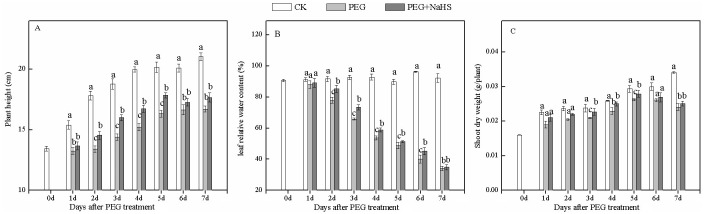
Effects of NaHS application on plant height, shoot weight, and leaf relative water content in wheat seedlings under drought stress. (A) Capital letters A, B and C stand for plant height, leaf relative water content, and shoot dry weight, respectively. (B) Different lower case letters indicate significant differences (*p*<0.05).

As shown in Fig 1A, the height of wheat seedlings decreased under drought stress conditions. NaHS pretreatment improved seedling height when plants were subjected to stress treatment, and the significant increase was found from the second day after stress (2 DAS) to 5 DAS. No significant difference was seen between the NaHS+PEG and PEG treatments at 6 and 7 DAS, which may be due to the protection function of H_2_S becoming weaker with prolonged drought stress times. We also noticed that the dry weight of wheat seedlings decreased in response to drought, but NaHS pretreatment significantly increased seedling weight at 3, 4, and 5 DAS (Fig 1C). For example, if the comparison was made against PEG, the NaHS+PEG treatment increased seedling dry weights at an average of 9.2%. The leaf relative water content (RWC) decreased under drought stress, and continued to decrease with increasing drought stress time (Fig 1B). Compared with the CK treatment, the leaf RWC in stressed plants (PEG) decreased by 14.9% and 63.1% at 2 DAS and 7 DAS, respectively. NaHS pretreatment increased leaf water status under drought stress and significant improvements were observed from 2 DAS to 6 DAS. If the comparison was made against the PEG treatment, the RWC in response to NaHS+PEG was increased by 11.4% at 3 DAS and at an overall average of 7.5%. These results indicated that NaHS pretreatment could improve plant water status in response to drought and alleviate the effects of drought stress.

### NaHS pretreatment reduces ROS accumulation in leaves and roots

The MDA contents of wheat leaves were shown in Table 2. Compared with the well-watered control (CK), the MDA contents in drought-stressed plants increased in response to drought stress. However, NaHS pretreatment reduced the leaf MDA contents in plants exposed to drought stress. The significant decline in leaf MDA content between the NaHS+PEG and PEG treatments was observed at 2 DAS and 4 DAS. The same trend was also observed in roots. In a comparison with the PEG treatment at 2 DAS, the root MDA contents in the corresponding NaHS pretreatments were decreased by 36.6%.

**Table 2:** Effects of NaHS pretreatment on MDA contents of wheat seedling leaves and roots (μmol/g Fw)^a^^,^ ^b^.

Tissue	Treatment	Days after drought stress							
		0	1	2	3	4	5	6	7
**Leaf**	**CK**	1.40	1.39c	1.38c	1.53b	1.33b	1.45a	1.17b	1.15a
	**PEG**		1.70a	2.43a	1.92a	1.56a	2.27a	1.68a	1.29a
	**NaHS+PEG**		1.68a	1.96b	1.87a	1.24b	2.08a	1.59ab	1.25a
**Root**	**CK**	0.46	0.51c	0.52c	0.51c	0.52c	0.50c	0.51c	0.52b
	**PEG**		0.62a	1.78a	1.92a	2.35a	0.75a	0.86a	0.89a
	**NaHS+PEG**		0.56b	1.13b	1.24b	2.03b	0.69b	0.76b	0.87a

^a^ Within each column, the same lower case letter indicates no significant difference between treatments (*p*<0.05);

^b^ Fw, Fresh weight.

As shown in Table 3, the leaf H_2_O_2_ contents were significantly increased in response to drought stress. Compared with the CK, the H_2_O_2_ content in leaves increased at an average of 94.4% in the PEG treatment. NaHS pretreatment decreased the H_2_O_2_ content during drought stress, but a significant difference was only observed from 4 to 6 DAS. We also noticed that exogenous H_2_S treatment significantly decreased the H_2_O_2_ content in roots exposed to drought stress. Compared with the PEG treatment, the root H_2_O_2_ contents were decreased at an average of 24.5% in the NaHS+PEG treatment. These results indicated that exogenous H_2_S can alleviate oxidative stress induced by drought stress.

**Table 3:** Effects of NaHS pretreatment on H_2_O_2_ contents of wheat seedling leaves and roots (μmol/g Fw)^a^^,^ ^b^.

Tissue	Treatment	Days after drought stress							
		0	1	2	3	4	5	6	7
**Leaf**	**CK**	1.57	1.45b	1.69b	1.55b	1.60c	1.91c	1.44c	1.35b
	**PEG**		2.32a	2.49a	2.65a	3.51a	3.79a	3.61a	2.91a
	**NaHS+PEG**		2.02b	1.88ab	2.26a	2.92b	3.25b	2.81b	2.77a
**Root**	**CK**	0.17	0.19b	0.22c	0.18b	0.17c	0.17c	0.19b	0.19b
	**PEG**		0.30a	0.37a	0.46a	0.59a	0.47a	0.35a	0.37a
	**NaHS+PEG**		0.21b	0.30b	0.38a	0.53b	0.33b	0.23b	0.24b

^a^ Within each column, the same lower case letter indicates no significant difference between treatments (*P*<0.05);

^b^ Fw, Fresh weight.

### NaHS pretreatment increases antioxidant capacity in wheat leaves and roots

**Fig 2:**
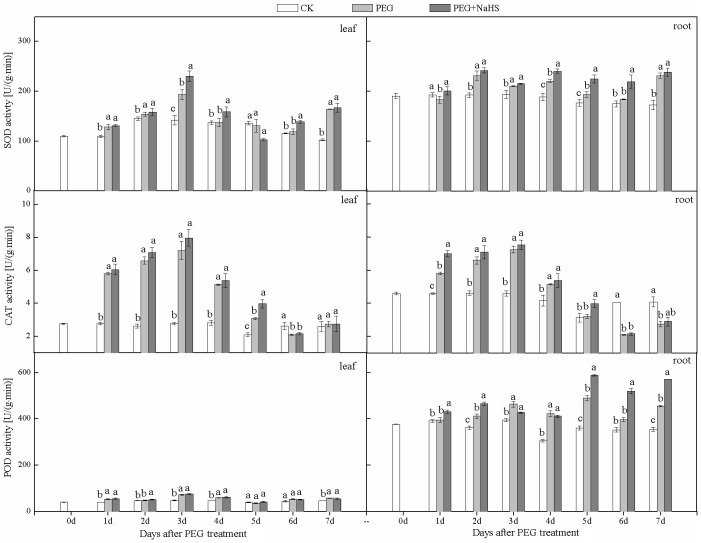
Effects of NaHS application on antioxidant enzyme activities in wheat leaves and roots in response to drought stress. Different lower case letters indicate significant differences (*p*<0.05).

As shown in Fig 2, the SOD activity in leaves exhibited an increasing trend during stress treatment and peaked at day 3 after treatment. NaHS pretreatment increased the SOD activity in leaves exposed to drought stress, and a significant difference was observed at 3, 4, and 6 DAS. A similar trend was also observed for CAT activity in leaves during the stress treatment. Results showed that the NaHS pretreatment significantly improved CAT activity at 5 DAS compared with the PEG treatment. The NaHS pretreatment also increased POD activity in leaves under drought, although the relative changes were lower than for SOD and CAT. For example, the average increase in POD activity between the NaHS+PEG and PEG treatments was only 4.1%, whereas the corresponding increase for CAT was 8.4%.

It was clear from our experiments that enzyme activities in roots were somewhat higher than in leaves, and this was especially true for POD, where the magnitude was quite high. Our results showed that SOD, CAT, and POD activities were increased in plants that were pretreated with NaHS at the different drought stress times. For example, compared with PEG alone, the activity of SOD in the NaHS+PEG treatment was significantly increased at 1, 4, 5, and 6 DAS, while CAT activity was significantly increased at 1 and 5 DAS.

### Expression profiling of genes involved in the ABA metabolic pathway in wheat leaves

**Fig 3:**
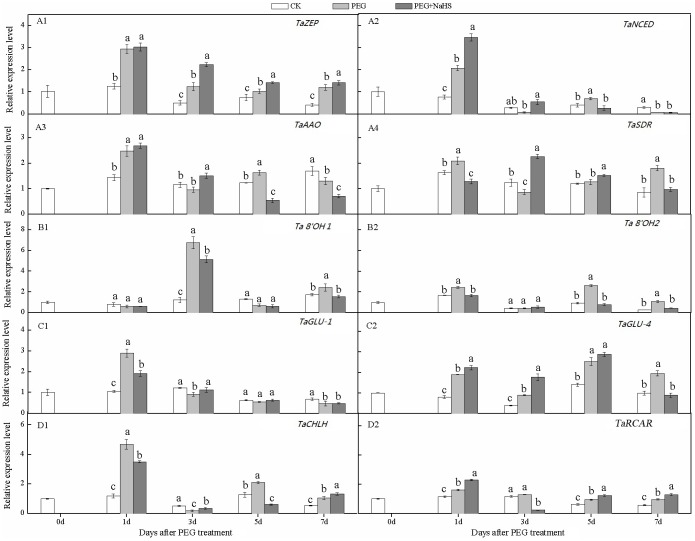
Effects of NaHS application on the relative expression levels of genes involved in the ABA metabolism pathway in wheat leaves in response to drought stress. Different lower case letters indicate significant differences (*p*<0.05).

As shown in Fig 3, the expression profiles of genes involved in the leaf ABA metabolic pathway varied widely in response to drought and exogenous NaHS application. The expression levels of *TaZEP* (Fig 3A1), *TaNCED* (Fig 3A2), *TaAAO* (Fig 3A3) and *TaSDR* (Fig 3A4) involved in leaf ABA biosynthesis exhibited increasing trend during stress treatment and peaked 1 day after treatment. NaHS pretreatment increased expression levels of these genes at different times after stress treatments. Compared with the PEG treatment, exogenous NaHS significantly increased the relative expression levels of *TaNCED* and *TaAAO* at 3 DAS, while the expression levels of *TaZEP* and *TaSDR* were significantly increased at 3, 5, and 7 DAS (except for *TaSDR* at 7 DAS). For example, the relative expression level of *TaZEP* for the NaHS+PEG treatment was increased at an average of 44.9% from 3 DAS to 7 DAS compared with PEG alone. The relative expression level of *TaNCED* in the NaHS+PEG treated plants was 1.7-fold higher than in the PEG treatment at 1 DAS. These results suggested that exogenous NaHS application has an effect on ABA biosynthesis during drought stress, and the relevant genes were up-regulated at different times in plants exposed to drought stress.

The relative expression levels of two genes coding for ABA catabolism enzymes (*Ta8’-OH1* and *Ta8’-OH2*), and two genes that encode ABA activation enzymes (*TaGLU1* and *TaGLU4*) were shown in Fig 3B1, 3B2, 3C1 and 3C2, respectively. As expected, drought treatment resulted in up-regulation of the genes involved in ABA catabolism at different times after stress treatment. However, NaHS pretreatment had no significant influence on the expression levels of *Ta8’-OH1* and *Ta8’-OH2* (except for *Ta8’-OH1* at 3 DAS), compared with the CK. The genes encoding ABA activation enzymes in leaves exhibited an increasing expression trend during stress treatment and peaked at 1 DAS and 5 DAS for *TaGLU1* and *TaGLU4*, respectively. We noticed that the effect of exogenous H_2_S treatment on *TaGLU4* was greater than on *TaGLU1*. Compared with PEG, the relative expression levels of *TaGLU4* in leaves treated with NaHS+PEG were 1.2-, 2.0-, and 1.1-fold higher at 1, 3, and 5 DAS, respectively.

The relative expression levels of genes coding for ABA receptors in leaves are shown in Fig 3D1 and 3D2. NaHS pretreatment significantly increased the expression levels of *TaRCAR* and *TaCHLH*, but at different times after stress treatment. Compared with PEG alone, the NaHS+PEG treatment increased the relative expression of *TaRCAR* by 42.6%, 28.7%, and 33.9% at 1, 5, and 7 DAS, respectively. Similarly, the up-regulation of *TaCHLH* in response to NaHS treatment was 1.9- and 1.3-fold higher than in the PEG treatment at 3 and 7 DAS, respectively. However, we also noticed that the expression levels of *TaCHLH* after NaHS pretreatment at 1 and 5 DAS were significantly lower than in the PEG treatment alone. This suggested that exogenous H_2_S can only up-regulate the expression of ABA receptor genes at certain times during a period of drought, and this up-regulation could potentially benefit ABA signal transduction.

### Expression profiles of genes involved in ABA metabolic pathways in wheat roots

**Fig 4:**
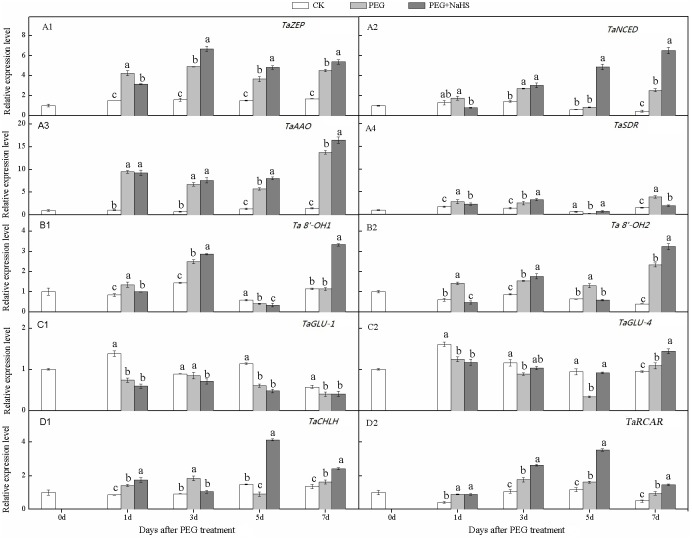
Effects of NaHS application on the relative expression levels of genes involved in the ABA metabolism pathway in wheat roots in response to drought stress. Different lower case letters indicate significant differences (*p*<0.05).

The expression profiles of genes involved in the ABA metabolic pathway in roots were shown in (Fig 4A, 4B, 4C and 4D). We found that the genes encoding enzymes for ABA biosynthesis in roots (*TaZEP*, *TaNCED*, *TaAAO*, and *TaSDR*) showed similar expression profiles in response to NaHS+PEG treatment. NaHS pretreatment significantly up-regulated the expression of *TaZEP*, *TaNCED*, *TaAAO*, and *TaSDR* in wheat roots. The expression of these genes in the NaHS+PEG treatment showed an increasing trend during the drought stress treatment and reached their peak values at 3 DAS for *TaZEP* and *TaAAO*, and at 7 DAS for *TaNCED* and *TaSDR*. The relative expression levels of *TaZEP*, *TaNCED*, *TaAAO*, and *TaSDR* in the NaHS+PEG treatment were approximately 1.3-, 3.2-, 1.5-, and 1.2-fold higher on average, respectively, than in the PEG treatment from 3 to 7 DAS. These results indicated that NaHS pretreatment can up-regulate the expression of ABA biosynthesis genes in roots.

NaHS pretreatment increased the relative expression levels of *Ta8’-OH1* (Fig 4B1) and *Ta8’-OH2* (Fig 4B2) in roots at 3 and 7 DAS, which was different from the situation in leaves. Compared with the PEG treatment at 3 DAS, the relative expression levels of *Ta8’-OH1* and *Ta8’-OH2* for the NaHS+PEG treatment were increased by 14.9% and 14.6%, respectively, while the relative expression levels of *Ta8’-OH1* and *Ta8’-OH2* were increased by 192.8% and 38.9% at 7 DAS. As shown in Fig 4C1 and 4C2, *TaGLU1* and *TaGLU4*, the relevant genes involved in ABA activation, did not show an obvious response to exogenous H_2_S during drought treatment (except for *TaGLU4* at 5 DAS). We noticed that there was a slight down-regulation of *TaGLU1* in response to drought stress, and there were no significant differences between the PEG and NaHS+PEG treatments (except for *TaGLU1* at 3 DAS).

The genes *TaRCAR* and *TaCHLH* that encode ABA receptors showed similar changes in response to the NaHS+PEG treatment. NaHS pretreatment significantly increased the expression levels of *TaRCAR* from 3 DAS to 7 DAS. Compared with PEG alone, the expression levels of *TaRCAR* in the NaHS+PEG treatment were increased by 48.9%, 117.7%, and 53.9% at 3, 5, and 7 DAS, respectively. For *TaCHLH*, the relative expression level in the NaHS pretreatment was almost 4.6-fold greater than that for PEG treatment alone at 5 DAS. These results showed that increasing the exogenous H_2_S concentration can possibly improve the relative expression levels of ABA receptor genes in wheat roots.

### ABA and H_2_S accumulation in leaves and roots of wheat seedlings

**Fig 5:**
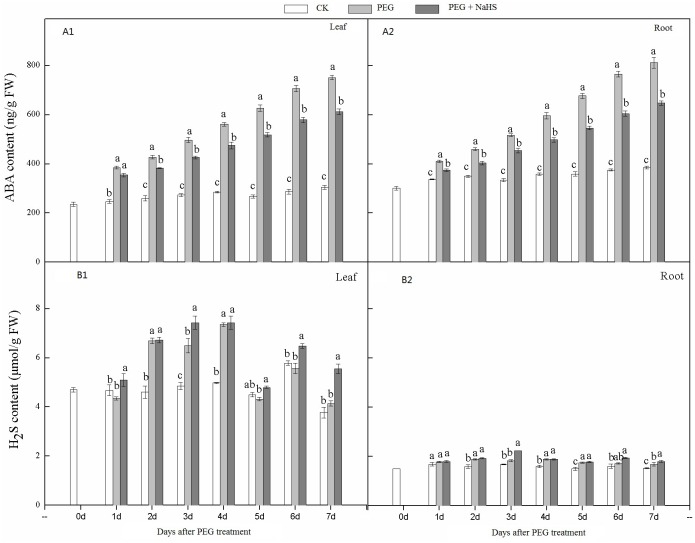
Effects of NaHS application on the H_2_S and ABA contents of wheat leaves and roots in response to drought stress. Different lower case letters indicate significant differences (*p*<0.05).

As shown in Fig 5A, the accumulation of ABA in the PEG and NaHS+PEG treatments in leaves and roots showed an increasing trend during the stress treatment, and peaked at 7 DAS. It is interesting that the content of ABA in leaves and roots in the NaHS+PEG treatment was lower than in the PEG treatment. We also noticed that the differences in ABA content between the PEG and NaHS+PEG treatments increased with extended stress times. For example, the differences in ABA levels in leaves between the PEG and NaHS+PEG treatments were 29.9 ng/g and 137.9 ng/g at 1 and 7 DAS, respectively. A similar trend was also observed in wheat roots. These results indicated that NaHS pretreatment can decrease the ABA content in leaves and roots during drought stress compared with drought stress alone.

Fig 5B showed that the H_2_S content in leaves tended to increase after drought stress treatments, and the highest values were observed at 4 DAS. Compared with PEG alone, the NaHS+PEG treatment significantly increased H_2_S content at all times except for 2 and 4 DAS. A similar trend for H_2_S content and stress time was also observed for wheat roots. Compared with PEG, the H_2_S content in roots for the NaHS+PEG treatment was significantly increased at 3, 6, and 7 DAS, indicating that exogenous H_2_S can increase the endogenous H_2_S levels in leaves and roots. The results also showed that the H_2_S content in leaves was higher than that in roots.

**Fig 6:**
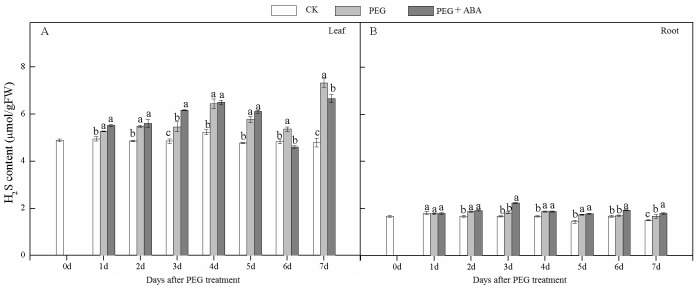
Effects of exogenous ABA application on the H_2_S and ABA content of wheat leaves and roots in response to drought stress. Different lower case letters indicate significant differences (*p*<0.05).

We also tested the H_2_S content of leaves and roots under drought stress conditions with exogenous application of ABA (Fig 6). The results showed that ABA application increased the endogenous H_2_S content in both leaves and roots under drought stress. Compared with the PEG treatment, the H_2_S content in roots in the ABA+PEG treatment increased at an average of 8.5% from 1 to 7 DAS. These results suggested that there may be cross talk between ABA and H_2_S.

## Discussion

### NaHS pretreatment alleviates drought stress by increasing antioxidant capacity

H_2_S is the third gaseous mediator after NO and CO, and plays an important role in various biological processes in plants. It has been reported that NaHS-treated plants grew better and exhibited increased survival rates compared to non-treated plants exposed to salt, drought, and cold stress [24]. In this work, exogenous H_2_S application resulted in significant increases in plant biomass and plant height, in agreement with a previous report by Zhang et al., who reported that spraying with the H_2_S donor (NaHS) increased both leaf and root biomass in soybean under continuous drought stress [13].

Previous reports have shown that H_2_S can alleviate damage in plants induced by different abiotic stresses via the improvement of antioxidant systems [14]. In this study, the antioxidant enzyme activities of SOD, CAT, and POD in leaves and roots increased in response to NaHS pretreatment compared with PEG treatment alone, indicating that H_2_S application can improve the antioxidant system to alleviate drought-induced oxidative damage. These results are in agreement with the report of Wang et al. [25]. However, Dawood et al. reported that CAT activity in barley increased while POD activity decreased with H_2_S application in alleviating Al-induced oxidative stress [11]. The discrepancy may be due to the different responses of different plants to oxidative stress, as well as the sample collection times. Here, we also found that CAT activity in roots decreased at 6 and 7 DAS. Zhang et al. suggested that NaHS can regulate antioxidant systems rapidly for the elimination of H_2_O_2_ caused by PEG exposure [26]. In present study, the results of increased antioxidant enzyme activity accompanied by a decrease in the MDA and H_2_O_2_ contents in wheat leaves and roots indicated that exogenous application of H_2_S can alleviate oxidative stress by increasing antioxidant systems.

### ABA metabolic pathway genes respond to exogenous H_2_S application

ABA plays an important role in drought tolerance in plants. Several studies have suggested that H_2_S could be involved in ABA signaling to induce stomatal closure in *Arabidopsis* [27, 28]. Li et al. reported that ABA-induced heat tolerance was enhanced by addition of NaHS [10]. In this study, the expression levels of ABA biosynthesis genes, *TaZEP*, *TaNCED*, *TaAAO*, and *TaSDR*, were up-regulated under drought stress in leaves and roots, which is in agreement with the report of Valliyodan and Nguyen [29]. These results also showed that exogenous application of NaHS improved the expression levels of ABA biosynthesis genes both in leaves and roots at different times during drought stress. The dynamic balance of ABA content in plants is regulated by biosynthesis and degradation. ABA 8′-hydroxylase (8′-OH) is the key enzyme in the ABA oxidative degradation pathway [15], and it is encoded by the cytochrome P450 monooxygenase gene CYP707A [30]. We found that drought stress caused up-regulation of *Ta8’-OH1* and *Ta8’-OH2* expression both in leaves and roots. Similar results were also found by Kushiro et al. [30] and Saito et al. [31]. Compared with drought stress, the expression levels of *Ta8’-OH1* and *Ta8’-OH2* in the roots were increased by NaHS pretreatment, but the expression levels in the leaf did not show the same patterns. This can be mainly attributed to the fact that the expression of these genes is regulated differently in the two tissues. Saito et al. reported that expression of the *CYP707A* genes, encoding ABA 8’-OH, was ubiquitous in various organs with different transcript accumulation levels; for example, in roots, *CYP707A1* and *CYP707A3* were moderately expressed, *CYP707A2* was expressed weakly, and no expression of *CYP707A4* was detected [31]. The conjugation of ABA with glucose results in ABA inactivation [17], but β-glucosidase can hydrolyze glucose-conjugated, biologically inactive ABA to produce active ABA [18]. In our study, *TaGLU1* and *TaGLU4*, which function in ABA reactivation, were up-regulated in the NaHS+PEG in the treatment in leaves while no obvious increase in expression was observed in roots. This may be due to differential expression of *TaGLU1* and *TaGLU4* in different organs. It is also possible that with the limited information in the present study, that there is another *TaGLU* gene involved in ABA activation that we did not find.

Plants need to adjust ABA levels constantly in response to changing physiological and environmental conditions, and bioactive ABA concentrations are maintained through a fine balance between biosynthesis and catabolism. In this work, we found that NaHS pretreatment up-regulated the expression of genes involved in ABA biosynthesis and ABA reactivation in leaves, whereas NaHS pretreatment up-regulated ABA biosynthesis and ABA catabolism genes in roots. Therefore, our results show that ABA participates in the drought tolerance induced by exogenous H_2_S, and that there are different responses in leaves and roots.

ABA receptors are responsible for ABA signal perception and transduction. Jin et al. [27] found that H_2_S is involved in the expression of genes for ABA receptors in leaves, and suggested that H_2_S might be involved in ABA signaling through an ABA receptor. In this work, we found that the expression levels of two genes encoding ABA receptor candidates in leaves and roots, *TaRCAR* and *TaCHLH*, were up-regulated in the NaHS+PEG treatment for a certain period of time. Similar results were also reported by Jin et al. [27], who found that the expression levels of ABA receptor candidate genes were reduced in response to H_2_S donor fumigation after 6 h, but that expression was up-regulated after 12 h for the receptor genes *ABAR*, *PYR1*, and *GTG1*. We also observed that the expression level of *TaCHLH* in leaves under NaHS+PEG treatment was significantly lower than in the PEG treatment at 1 DAS and 5 DAS, while *TaRCAR* was down-regulated in the NaHS+PEG treatment at 3 DAS when compared with PEG alone (Fig 3). One possible explanation is that these genes respond differently to exogenous substances and the length of the drought stress period. Compared with the corresponding PEG treatment alone, *TaRCAR* in the NaHS + PEG treatment showed significant up-regulated expression early (1 DAS), whereas *TaCHLH* tended to be highly expressed later (7 DAS). Another reason may be that the expression levels of these genes are related to the concentration of ABA or other chemical signals. The other possibility is that there are different regulatory mechanisms for the different receptors. Since the RNA binding protein FCA was reported to be an ABA receptor [32], several other ABA receptor candidates have been identified [33, 34]. In this study, only two ABA receptor candidate genes, *TaCHLH* and *TaRCAR*, were tested. Other ABA receptor candidate genes may exist, and further experiments should be conducted to explore the underlying relationships between H_2_S regulation and ABA receptors.

### Crosstalk between H_2_S and ABA

Li and Jin suggested that there are interactions between H_2_S and ABA in the acquisition of heat tolerance in tobacco [10]. In the present study, ABA content was higher in the PEG treatment than it was in the NaHS+PEG treatment. As mentioned above, the ABA contents are regulated differently in leaves and roots. The lower levels of ABA in the NaSH+PEG treatment may be due to several reasons. First, low ABA concentrations can induce signal transduction and increase drought tolerance. Second, the balance of ABA is important for certain plant physiological processes, and lower ABA contents would maintain normal plant growth. Third, higher expression levels of ABA receptor genes could increase perception and signal transduction even at lower ABA contents. We also noticed that the H_2_S contents in leaves and roots all increased in the NaHS+PEG treatments. The elevated H_2_S levels may play a similar role to ABA, just as Jin et al. [27] suggested, because ABA regulates many physiological processes, and the functions of H_2_S are similar to ABA in certain respects. The findings of Li and Jin [10] indicated that ABA can induce the accumulation of endogenous H_2_S, which is a new downstream gaseous signal molecule in ABA-induced heat tolerance in tobacco. In our supplementary experiments, the H_2_S contents of wheat leaves and roots were all found to increase when ABA was applied during drought treatment, indicating that there is complex crosstalk between these two signal molecules. However, it seems that there is no simple upstream or downstream linear relationship between these two signal molecules [27]. On the basis of all of the above-mentioned studies, we suggested that the alleviation of drought stress by H_2_S, at least in part, involves the ABA signaling pathway.

## Supporting Information

S1 File Supporting information about data. (XLS)

## Abbreviations

AAO: abscisic aldehyde oxidase
ABA: abscisic acid
CAT: catalase
CHLH: H subunit of Mg-chelatase
FW: fresh weight
GLU: β-glucosidases
H_2_S: Hydrogen sulfide
MDA: Malondialdehyde
NaHS: Sodium hydrosulfide
NCED: 9-cis-epoxycarotenoid dioxygenase
POD: peroxidase
RCAR: the regulatory component of ABA
SDR: short-chain dehydrogenase
SOD: superoxide dismutase
ZEP: zeaxanthin epoxidase
